# Causal-aware reliability assessment of single-channel EEG for transformer-based sleep staging

**DOI:** 10.3389/fnins.2025.1670124

**Published:** 2025-10-08

**Authors:** Yongkang Hu, Xiangbo Yang, Yunhan Xu, Jingpeng Sun

**Affiliations:** School of Artificial Intelligence, Anhui University, Hefei, China

**Keywords:** sleep staging, single-channel EEG, causal learning, transformer, classification reliability

## Abstract

Single-channel EEG-based sleep staging methods are well-suited for wearable applications in home environments, offering a practical solution to reduce the diagnostic burden on clinical institutions and address the growing demand for large-scale sleep monitoring. However, its reliability remains a critical concern compared to multi-channel polysomnography (PSG) used in clinical settings. To address this, we propose a Transformer-based sleep staging model and conduct a systematic investigation into the causal-inspired analysis between EEG channel selection and staging reliability. Our experiments reveal that electrodes positioned over the central brain region yield significantly higher accuracy, macro-F1, and consistency in sleep stage classification compared to those located in frontal or occipital regions. These findings provide causal insights into the spatial determinants of perceptual reliability in EEG-based sleep monitoring, supporting the design of robust wearable systems.

## 1 Introduction

Sleep staging plays a vital role in evaluating sleep quality, diagnosing sleep disorders, and assessing both physical and mental health. Different sleep stages reflect distinct physiological states, characterized by complex spatio-temporal interactions among various systems. In clinical practice, polysomnography (PSG) is the standard approach for sleep monitoring, simultaneously recording multiple physiological signals, including electroencephalography (EEG), electrooculography (EOG), electrocardiography (ECG), and electromyography (EMG), across different regions of the brain and body. This multimodal setup captures temporal variations across spatially distributed channels. Sleep experts manually score each 30-second epoch by analyzing the characteristics of individual signals and their interrelations, following standardized guidelines such as the American Academy of Sleep Medicine (AASM) Scoring Manual (Berry et al., 2017) or the Rechtschaffen and Kales (R&K) manual (Hobson, 1969). The 30-second epoch length is the clinical standard widely used in sleep staging. However, manual annotation of a single overnight PSG recording typically requires approximately two hours of expert labor. With the increasing need for accurate sleep assessment, diagnosis, and long-term monitoring in home environments (Mikkelsen et al., 2019), manual scoring has become increasingly impractical due to its labor-intensive and time-consuming nature. Moreover, the process is highly dependent on individual expertise, rendering it susceptible to human error and inconsistencies. These limitations have motivated the development of automatic sleep staging methods, with recent efforts increasingly focused on causal-aware and self-supervised learning paradigms to improve generalization and interpretability.

Over the past two decades, automatic sleep staging methods have been broadly categorized into two main types: (1) traditional machine learning approaches and (2) deep learning-based approaches. Traditional machine learning methods typically involve a two-stage pipeline. In the first stage, hand-crafted features are extracted from physiological signals–either directly, such as temporal and frequency-domain features, or indirectly through signal processing techniques such as signal decomposition (Hu et al., 2021; Cheng et al., 2024). In the second stage, classifiers such as support vector machines (SVM), k-nearest neighbors (kNN), random forests, and decision trees are employed to categorize the extracted features and perform sleep stage classification (Satapathy et al., 2021; Sekkal et al., 2022). However, the performance of traditional methods heavily relies on expert-defined features and empirical thresholds, resulting in limited generalization capability and poor adaptability in real-world scenarios.

With the advent of deep learning, its powerful feature representation and end-to-end learning capabilities have significantly advanced automatic sleep staging. Numerous deep learning-based methods have been proposed to improve the accuracy and efficiency of sleep stage classification (Phan et al., 2021; Phan and Mikkelsen, 2022; Niknazar and Mednick, 2024). For example, Ji et al. (2023) proposed 3DSleepNet, a model based on 3D convolutional neural networks (CNNs), which simultaneously captures spatial, spectral, and temporal dependencies in multi-channel physiological signals. Compared to conventional 2D CNNs, the 3D architecture enables more effective modeling of dynamic signal evolution across time. Phan et al. (2021) introduced XSleepNet, a sequence-to-sequence model that processes both raw multi-modal signals (EEG, EOG, EMG) and their time-frequency representations, achieving promising results. Additionally, Niknazar and Mednick (2024) employ a bidirectional long short-term memory (Bi-LSTM) network combined with a signal decomposition mechanism to enhance the interpretability of feature learning for automatic sleep stage classification.

Despite the promising performance of existing methods, many rely on large volumes of input data–often requiring multiple modalities or multi-channel EEG recordings. As a result, these approaches are better suited for clinical environments rather than portable, wearable applications in home settings. To bridge this gap, researchers have increasingly focused on developing single-channel EEG-based sleep staging methods (Zaman et al., 2025).

For example, Supratak et al. (2017) proposed DeepSleepNet, a convolutional neural network (CNN)-based model that extracts local features from single-channel EEG signals for sleep staging. Perslev et al. (2019) introduced U-Time, a fully convolutional network inspired by the U-Net architecture, which maps single-channel EEG signals to a high-dimensional space and then projects them back to a lower-dimensional representation. To incorporate temporal dependencies, Supratak et al. later developed TinySleepNet (Supratak and Guo, 2020), a hybrid model that first extracts local features using CNNs and subsequently models temporal information with recurrent neural networks (RNNs), effectively combining both types of information for sleep staging. With the growing success of Transformer models in time-series modeling, Phan et al. (2022) introduced SleepTransformer, the first sleep staging model based on the Transformer architecture, and achieved strong performance using single-channel EEG data. Wang et al. (2025) proposed EfficientSleepNet, a lightweight architecture for single-channel EEG-based sleep staging that incorporates depthwise separable convolutions, grouped convolutions, channel reordering, and a novel channel attention mechanism to enhance efficiency and performance.

Although these single-channel methods have demonstrated encouraging results, they often overlook important design considerations, such as the selection of EEG channels. For instance, DeepSleepNet uses Fpz-Cz and Pz-Cz, U-Time adopts Fpz-Cz and C3-A2, and SleepTransformer utilizes Fpz-Cz and C4-A1, but none of these studies provide a systematic justification for their channel choices. Furthermore, they do not explore how staging performance varies across different channels, nor do they investigate potential limitations of single-channel approaches across specific sleep stages. These aspects are critical for the practical deployment of wearable single-channel sleep monitoring systems. To address these gaps, this study proposes a causal-aware Transformer-based sleep staging model that integrates convolutional neural networks (CNNs) with a Transformer architecture. The model is designed for single-channel EEG-based sleep staging and further enhanced by incorporating EOG signals to capture multimodal interactions. We systematically investigate the electrode-driven causal influence of EEG channel selection on staging performance and analyze the variability of classification accuracy across different sleep stages. Our work contributes to the development of personalized, interpretable, and deployable sleep monitoring systems, aligning with the broader goals of causal self-supervised learning and perception science.

The contributions of this paper are as follows:

- We propose a novel sleep staging model that integrates CNNs with a Transformer architecture, enabling effective feature extraction and temporal modeling from single-channel EEG signals. The model is further enhanced by incorporating EOG signals to capture cross-modal causal interactions.
- We conduct a systematic investigation into the causal impact of EEG channel selection on sleep staging performance, addressing a critical yet underexplored aspect of model design.
- We analyze the limitations and variability of single-channel EEG-based sleep staging across different sleep stages, providing insights into the reliability, interpretability, and generalizability of such models in real-world applications.

## 2 Methodology

**Figure 1:**
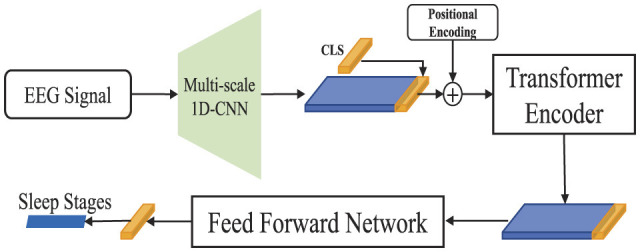
The architecture of SingleSleep. The model takes in raw single-channel EEG signals and extracts features using multi-scale 1D-CNN and transformer encoders. Sleep staging is then performed using the final classification layer.

**Figure 2:**
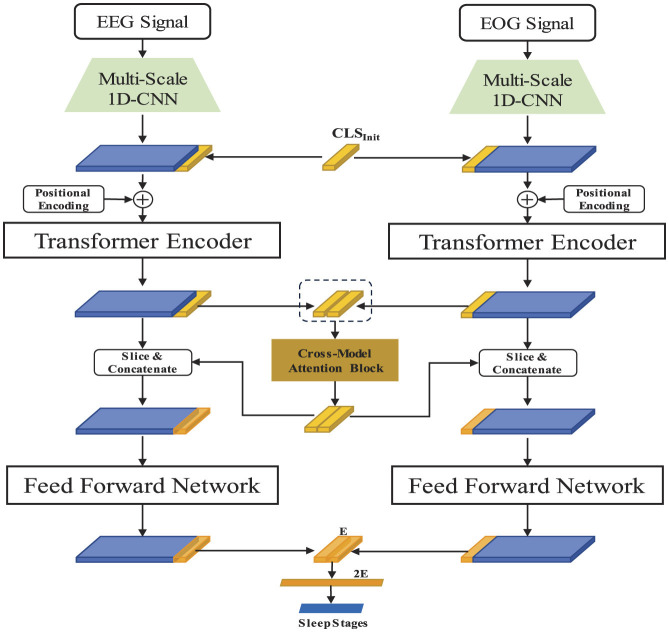
The SingleSleepPlus architecture integrates raw single-channel EEG and EOG signals as input. Each modality's features are independently extracted using multi-scale 1D-CNN and transformer encoders, then fused via a cross-modal attention block. Sleep staging is subsequently accomplished through the final classification layer.

In this section, we delve into the detailed introduction of our proposed models, SingleSleep and SingleSleepPlus. The SingleSleep model is tailored to utilize a single-channel EEG signal as its sole input, focusing exclusively on leveraging this singular bio-signal for sleep staging purposes. In contrast, the SingleSleepPlus model integrates both single-lead EEG and EOG signals as inputs, with the primary objective of enhancing sleep staging performance by augmenting the model with the additional EOG modality. We assess the efficacy of SingleSleep by comparing its classification outcomes with those of SingleSleepPlus, thereby elucidating the inherent limitations associated with relying solely on EEG signals for sleep staging. The architecture of the proposed SingleSleep and SingleSleepPlus are shown in Figures 1, 2.

### 2.1 Problem statement

The task of single-channel EEG sleep staging, derived from a complete nocturnal clinical EEG recording, is framed as a sequential, multi-class classification challenge. A full-night recording encompasses numerous epochs, each spanning 30-second, and is categorized into one of the five distinct sleep stages: WAKE, N1, N2, N3, or REM.

Accordingly, the training dataset, denoted by a set of N instances, comprises 30-second epochs with corresponding labels ${\left\{x_{i},y_{i}\right\}}_{i=1}^{N}$, where each {*x*_*i*_, *y*_*i*_} is drawn from the product space *X*×*Y*. The space *X*, represented as *X*∈ℝ^*T*×*C*^encapsulates the input features within an epoch, with *C* encompassing the EEG (and EOG) modalities present in the recorded signals. The label space *Y* is characterized by the set {WAKE, N1, N2, N3, and REM}, aligning with the respective sleep stages.

In formal terms, the sleep staging problem is construed as the learning process of an artificial neural network, denoted as *F*, which is predicated on a transformer-based architecture. The network *F* is designed to discern the contextual relationships within the input sequence of sleep epochs *X* and to map these sequences onto the corresponding sleep stage representation Ŷ, The output Ŷ, taking values in the set {0, 1, 2, 3, 4}, corresponds to the sleep stages WAKE, N1, N2, N3, and REM, respectively.

### 2.2 Model components

The proposed model comprises three major components: multi-scale 1D-CNN, Transformer encoder, and Cross-modal fusion module.

#### 2.2.1 Multi-scale 1D-CNN

Sleep stage data is embedded within the EEG signals, encompassing both local and global informational layers. The local features pertain to characteristic sleep waveforms such as slow waves, sleep spindles, and K-complexes, while the global features relate to the collective information among these waveforms across an entire EEG epoch. To effectively extract sleep stage information from EEG, it is essential to analyze and integrate both local and global informational content. Capitalizing on the proficiency of traditional CNNs in capturing local features, we adopt CNN as our extractor of local features. To also encompass global features, we enhance CNN's receptive field by incorporating convolutional kernels of diverse sizes, thereby augmenting its capacity to extract global characteristics. Prior research supports the efficacy of multi-scale CNNs for this objective. Consequently, we employ the multi-scale 1D-CNN module delineated in Pradeepkumar et al. (2022) as the preliminary feature extractor for EEG analysis. The multi-scale 1D-CNN module comprises three branches, each employing convolutional layers with varying kernel sizes: one branch uses a 1D-CNN with a kernel size of 50, another employs two 1D-CNNs with kernel sizes of 25 and 2, respectively, and the third branch utilizes three 1D-CNNs with kernel sizes of 5, 5, and 2. After each 1D convolutional operation, a LeakyReLU activation function is applied. The multi-scaled features are subsequently standardized through batch normalization. The aggregated feature representations are then concatenated along the embedding dimension, followed by a subsequent 1D-CNN with a kernel size of 1, which is itself followed by LeakyReLU activation and another round of batch normalization. It should be highlighted that the convolutional process utilizes non-overlapping windows that are 0.5 seconds (sampling rate: 200 Hz) in length. That is, given a single channel input sequence $X_{c}\in {\mathbb{R}}^{T\times 1}$ of length *T*, it is mapped into a feature space of $X_{c}^{\prime }\in {\mathbb{R}}^{\left(T/\left(0.5\times f_{s}\right)\right)\times E}$, where *c*∈*C*, *f*_*s*_ is the sampling frequency and *E* is the embedding size.

#### 2.2.2 Transformer encoder and cross-modal fusion module

The self-attention mechanism serves as the cornerstone of the transformer encoder. Here, we elucidate the principles of self-attention and the primary training process, utilizing SingleSleepPlus as an exemplar.

Upon receiving the output feature $X_{c}^{\prime }$ from the multi-scale 1D-CNN block for each modality *c*, a trainable *CLS*_*Init*_ vector in ℝ^1 × *E*^–similar to the approach advocated in ViT (Dosovitskiy et al., 2020)—is randomly initialized and appended to the output of the multi-scale 1D-CNN block along the time axis. Following the methodology delineated in seminal work (Vaswani et al., 2017), positional encodings are incorporated into the concatenated vector, which is subsequently fed into the transformer encoder to discern the relationships among all-time steps within the modality.

Given the input features *X*_*t*_ of the Transformer encoder, self-attention learns three representation matrices $W_{Q}\in {\mathbb{R}}^{d\times d_{q}}$, $W_{K}\in {\mathbb{R}}^{d\times d_{k}}$, and $W_{V}\in {\mathbb{R}}^{d\times d_{v}}$. These matrices are utilized to derive the query *Q* = *X*_*t*_*W*_*Q*_, key *K* = *X*_*t*_*W*_*K*_, and value *V* = *X*_*t*_*W*_*V*_, facilitating the computation of global attention as depicted by the formula:


(1) $$\begin{array}{l} \text{attention}=\text{Softmax}\left(\frac{QK^{T}}{\sqrt{d_{q}}}\right)V \end{array}$$

From the output of each modality, only the vector representation corresponding to the *CLS*_*Init*_ vector is extracted. This vector encapsulates all intra-modal temporal information. Subsequently, the class token vectors from each modality's output are amalgamated and utilized as input to the cross-modal fusion module. The cross-modal fusion module, akin to a simplified transformer encoder, facilitates the exchange and fusion of class information between modalities via self-attention. Finally, the merged class tokens from each modality are combined with their respective features and passed through a Feed-Forward Network layer. This is followed by the integration of class tokens from each modality to facilitate sleep staging.

## 3 Experiments

### 3.1 Dataset

To evaluate our proposed model, we utilized EEG and EOG signals from the ISRUC-Sleep dataset (Khalighi et al., 2016), comprising three subsets: ISRUC-S1, ISRUC-S2, and ISRUC-S3. ISRUC-S1 contains recordings from 100 subjects with various sleep-related disorders, ISRUC-S2 includes recordings from 8 subjects with mild sleep problems across two sessions and dates, and ISRUC-S3 comprises recordings from 10 healthy subjects. For our analysis, we focused on the healthy subset, ISRUC-S3, with subjects ranging in age from 30 to 58 years All recordings followed the international 10-20 standard electrode placement. Table 1 presents the number of epochs for each sleep stage. Each recording consists of 19 channels, including EOG, EEG, EMG, ECG, snore, and body position. The sampling rate for EOG, EEG, and EMG signals is 200Hz. Our sleep staging utilized six EEG channels (F3-A2, C3-A2, O1-A2, F4-A1, C4-A1, and O2-A1) and two EOG channels (LOC-A2 and ROC-A1) from PSG recordings. Annotations adhere to the AASM standard, encompassing five sleep stages (WAKE, N1, N2, N3, REM), and are provided by two professional experts. Importantly, our methodology utilizes raw EEG signals without additional feature extraction, such as conversion into time-frequency images. Additionally, no data augmentation is applied during training, which ensures full reproducibility of the results.

**Table 1:** Details of the dataset used in our experiments.

**Datasets**	**W**	**N1**	**N2**	**N3**	**REM**
ISRUC-S3	1,674	1,217	2,616	2,016	1,066
(Percentage)	19.50%	14.17%	30.46%	23.47%	12.40%

### 3.2 Evaluation criteria

We illustrate the model's performance using various evaluation metrics, including accuracy (ACC), macro-averaged F1-score (MF1), sensitivity (Sens.), and specificity (Spec.). ACC provides a straightforward measure of the proportion of correctly predicted samples out of the total sample count. MF1, representing the harmonic mean of precision (Pr) and recall (Re), holds particular importance in imbalanced multi-classification tasks like sleep staging. Below are the equations for each evaluation metric:


(2) $$\begin{array}{ccc} ACC & = & \frac{1}{N}\overset{K}{\underset{i=1}{\sum }}TP_{i} \end{array}$$

(3) $$\begin{array}{ccc} MF1 & = & \frac{1}{K}\overset{K}{\underset{i=1}{\sum }}\frac{2\times {\text{Pr}}_{i}\times {\text{Re}}_{i}}{{\text{Pr}}_{i}+{\text{Re}}_{i}} \end{array}$$

(4) $$\begin{array}{ccc} Sensitivity & = & \frac{1}{K}\overset{K}{\underset{i=1}{\sum }}\frac{TP_{i}}{TP_{i}+FN_{i}} \end{array}$$

(5) $$\begin{array}{ccc} Specificity & = & \frac{1}{K}\overset{K}{\underset{i=1}{\sum }}\frac{TN_{i}}{TN_{i}+FP_{i}} \end{array}$$

where True Positives (*TP*_*i*_), False Positives (*FP*_*i*_), and True Negatives (*TN*_*i*_) denote the correct or incorrect categorizations for the *i*-th class. *Pr*_*i*_ = *TP*_*i*_/(*TP*_*i*_+*FP*_*i*_) and *Re*_*i*_ = *TP*_*i*_/(*TP*_*i*_+*FN*_*i*_). *N* denotes the total number of samples, while *K* indicates the number of sleep stage classes. Furthermore, we utilized class-specific F1-score (class-specific MF1) to assess the model's performance. This metric treats each sleep stage as the positive class while regarding the other four stages as the negative class. The class-specific MF1 is calculated akin to binary classification, as depicted in Equation 3, without any averaging.

### 3.3 Training setup

For network training, we employed the Adam optimizer with a learning rate of 0.001, setting β1 and β2 to 0.9 and 0.999, respectively. A batch size of 32 was used during training. Categorical cross-entropy served as the loss function for the 5-class classification task, where the class weights were defined as 1, 2, 1, 1, 2, corresponding to Wake, N1, N2, N3, and REM, respectively, to address the data imbalance. Regarding the transformer encoder and cross-modal fusion module, we maintained 8 attention heads and 128 hidden units in the feed-forward layer. The PyTorch framework was utilized for model implementation, and training was conducted on an Nvidia 3090 GPU equipped with 24 GB of memory.

## 4 Results

### 4.1 Comparison among different channels

To investigate the impact of different EEG channels on the performance of single-channel EEG sleep staging models, experiments were conducted using various channels, as detailed in Table 2. The A1 and A2 lobe represent the left and right reference lobe placed on the earlobes, respectively, while F3 and F4 denote lobes from the left and right frontal lobes. Similarly, C3 and C4 correspond to lobes from the left and right central lobes, and O1 and O2 indicate lobes from the left and right occipital lobes. From the experimental results, it is evident that C3 and C4 achieved the highest performance, with accuracies of 77.16% and 76.79%, and MF1 scores of 71.61% and 69.98%, respectively, outperforming the other four channels. Furthermore, the frontal lobe channels (F3 and F4) exhibited superior performance compared to the occipital lobes channels, indicating that the occipital lobe channels performed the poorest among all six channels. This suggests that selecting occipital lobe channels for single-channel EEG sleep staging tasks may not be optimal, implying that results from central lobe channels are more reliable, followed by frontal lobe channels, while occipital lobe channels exhibit the lowest reliability.

**Table 2:** Performance comparison among different channels.

**Dataset**	**Channel**	**Overall metrics**				**Class-wise MF1**				
		**Acc**.	**MF1**	**Sens**.	**Spec**.	**W**.	**N1**	**N2**	**N3**	**REM**
ISRUC-S3	F3-A2	75.30	66.85	68.61	93.65	89.73	47.56	75.93	86.51	34.53
		F4-A1	72.96	63.25	64.21	93.03	90.48	40.81	76.25	82.30	26.43
		C3-A2	77.16	71.61	74.14	94.43	92.51	47.55	75.95	88.57	53.49
		C4-A1	76.79	69.98	72.76	94.26	91.80	39.00	78.29	88.68	52.14
		O1-A2	72.34	66.73	70.87	93.14	89.07	38.14	66.23	75.86	64.36
		O2-A1	70.75	63.25	64.59	92.49	86.85	34.16	71.48	78.26	45.47
		F3-A2 & LOC^*^-A2	80.01	76.87	**78.55**	94.96	90.83	52.70	78.47	88.74	**73.64**
		F3-A2 & ROC^*^-A1	78.30	73.13	74.78	94.47	91.39	46.21	77.00	**90.33**	60.70
		F4-A1 & LOC-A2	77.70	73.45	75.03	94.43	89.13	50.03	78.35	88.93	60.79
		F4-A1 & ROC-A1	77.44	71.81	74.31	94.39	91.27	42.83	74.81	88.63	61.49
		C3-A2 & LOC-A2	80.28	75.49	76.64	95.02	92.32	48.14	80.16	90.12	66.69
		C3-A2 & ROC-A1	79.37	72.51	73.13	94.67	**92.84**	47.52	78.82	88.19	55.20
		C4-A1 & LOC-A2	**81.50**	**77.15**	78.10	**95.32**	91.17	**53.37**	**81.55**	89.46	70.20
		C4-A1 & ROC-A1	78.58	74.56	76.35	94.65	90.86	51.17	78.04	87.52	65.21
		O1-A2 & LOC-A2	77.91	73.11	75.11	94.45	88.03	45.06	77.82	88.52	66.11
		O1-A2 & ROC-A1	75.96	72.01	74.48	93.96	89.77	47.22	74.63	82.12	66.31
		O2-A1 & LOC-A2	78.61	72.34	73.47	94.42	88.69	39.86	80.33	86.95	65.85
		O2-A1 & ROC-A1	71.76	64.72	65.66	92.72	87.88	46.58	72.20	71.55	45.40

^*^LOC-A2: Left eye movements; ROC-A1: Right eye movements. The A1 and A2 are placed in the left and right ear-lobes. The bold values indicates the best experimental results.

However, while the central lobe channels generally achieve the highest classification accuracy overall, it does not imply that they consistently perform best in all sleep stages. As shown in Table 2, each channel exhibits variations in classification performance across different sleep stages. Specifically, the frontal lobe channels demonstrate the best identification performance for the N1 stage, while the central lobe channels exhibit relatively good classification efficacy for the N2 and N3 stages, and the occipital lobe channels provide more reliable identification for the REM stage. Therefore, when conducting specific analyses targeting particular sleep stages, selecting channels that demonstrate optimal performance in capturing features relevant to the corresponding sleep stage, rather than those with the best overall performance, yields more reliable results.

### 4.2 Limitations of single-channel EEG model

**Figure 3:**
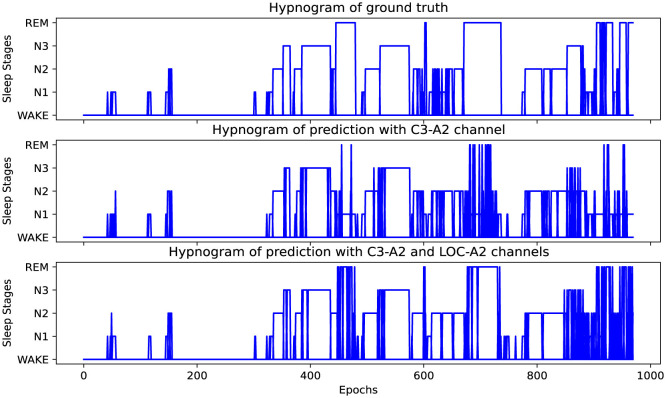
Hypnograms of one subject of the ISRUC-S3 dataset (subject 8). **Top row**: ground truth of sleep stages; **middle row**: stage classification result with C3-A2 channel, accuracy is 76.29%, and macro F1-score is 66.01 for this subject; **bottom row**: stage classification result with C3-A2 and LOC-A2 channels. Accuracy is 84.43%, and macro F1-score is 79.97 for this subject.

To investigate the limitations of single-channel EEG in sleep staging, we enhanced the performance by introducing EOG modality information and compared it with the performance of the single-channel EEG to analyze the performance of the single-channel EEG sleep staging model in identifying each sleep stage. As shown in Table 2, the overall accuracy of the model increased by 4.71% and the MF1 increased by 7.17% after adding the EOG modality (for C4-A1 channel). The main reason for this improvement is the enhancement of the classification performance for the REM stage, which is prominently demonstrated in the example results depicted in Figure 3. Meanwhile, the N1 stage also experienced a noticeable improvement, while the impact on other sleep stages was relatively minor. This is mainly because the eye movement information during the N1 and REM sleep stages provides significant complementary information, and the scoring rules for the N1 and REM stages also include definitions related to EOG. This indicates that SingleSleepPlus accurately captures information from EEG and EOG and successfully integrates the information from both modalities. It also suggests that the single-channel EEG sleep staging model still needs improvement in recognizing REM and N1 stages, especially in capturing features related to the REM stage. However, it demonstrates relatively stable performance in the N3 sleep stage, showing a relatively high level of reliability.

## 5 Conclusion and future work

The reliability analysis of portable wearable single-channel EEG sleep staging approaches for home use scenarios is crucial for objectively assessing sleep quality. However, there is currently a lack of study in this field. To address this gap, in this paper, we propose two different models to investigate the reliability of single-channel EEG in sleep staging. On one hand, we analyze the differences in reliability among channels in single-channel sleep staging tasks. On the other hand, we study the reliability of single-channel EEG sleep staging methods in identifying features of different sleep stages.

In future research, we will investigate more channels to provide a more systematic and comprehensive reliability analysis of single-channel EEG sleep staging methods, thus laying the groundwork for wearable sleep staging applications.

## Data Availability

The original contributions presented in the study are included in the article/supplementary material, further inquiries can be directed to the corresponding author.
